# Scalp-Mounted Electrical Impedance Tomography of Cerebral Hemodynamics

**DOI:** 10.1109/JSEN.2022.3145587

**Published:** 2022-01-21

**Authors:** Taweechai Ouypornkochagorn, Nataša Terzija, Paul Wright, John L. Davidson, Nick Polydorides, Hugh McCann

**Affiliations:** Faculty of Engineering, Srinakharinwirot University, Bangkok 10110, Thailand; Arriver Software GmbH, 85716 Unterschleißheim, Germany; Department of Electrical and Electronic Engineering, The University of Manchester, Manchester M13 9PL, U.K; School of Engineering, The University of Edinburgh, Edinburgh EH9 3JL, U.K

**Keywords:** Cerebral, EIT, electrical impedance tomography, SNR, hemodynamic, REG, rheoencephalogram, transient hyperemic response, THR

## Abstract

An Electrical Impedance Tomography (EIT) system has been developed for dynamic three-dimensional imaging of changes in conductivity distribution in the human head, using scalp-mounted electrodes. We attribute these images to changes in cerebral perfusion. At 100 frames per second (fps), voltage measurement is achieved with full-scale signal-to-noise ratio of 105 dB and common-mode rejection ratio > 90 dB. A novel nonlinear method is presented for 3-D imaging of the difference in conductivity distribution in the head, relative to a reference time. The method achieves much reduced modelling error. It successfully localizes conductivity inclusions in experimental and simulation tests, where previous methods fail. For > 50 human volunteers, the rheoencephalography (REG) waveform is observed in EIT voltage measurements for every volunteer, with peak-to-peak amplitudes up to approx. 50 *μ*V_rms_. Images are presented of the change in conductivity distribution during the REG/cardiac cycle, at 50 fps, showing maximum local conductivity change of approx. 1% in grey/white matter. A total of 17 tests were performed during short (typically 5s) carotid artery occlusions on 5 volunteers, monitored by Transcranial Doppler ultrasound. From EIT measurements averaged over complete REG/cardiac cycles, 13 occlusion tests showed consistently decreased conductivity of cerebral regions on the occluded side, and increased conductivity on the opposite side. The maximum local conductivity change during occlusion was approx. 20%. The simplicity of the carotid artery intervention provides a striking validation of the scalp-mounted measurement system in imaging cerebral hemodynamics, and the REG images indicate its unique combination of sensitivity and temporal resolution.

**Figure:**
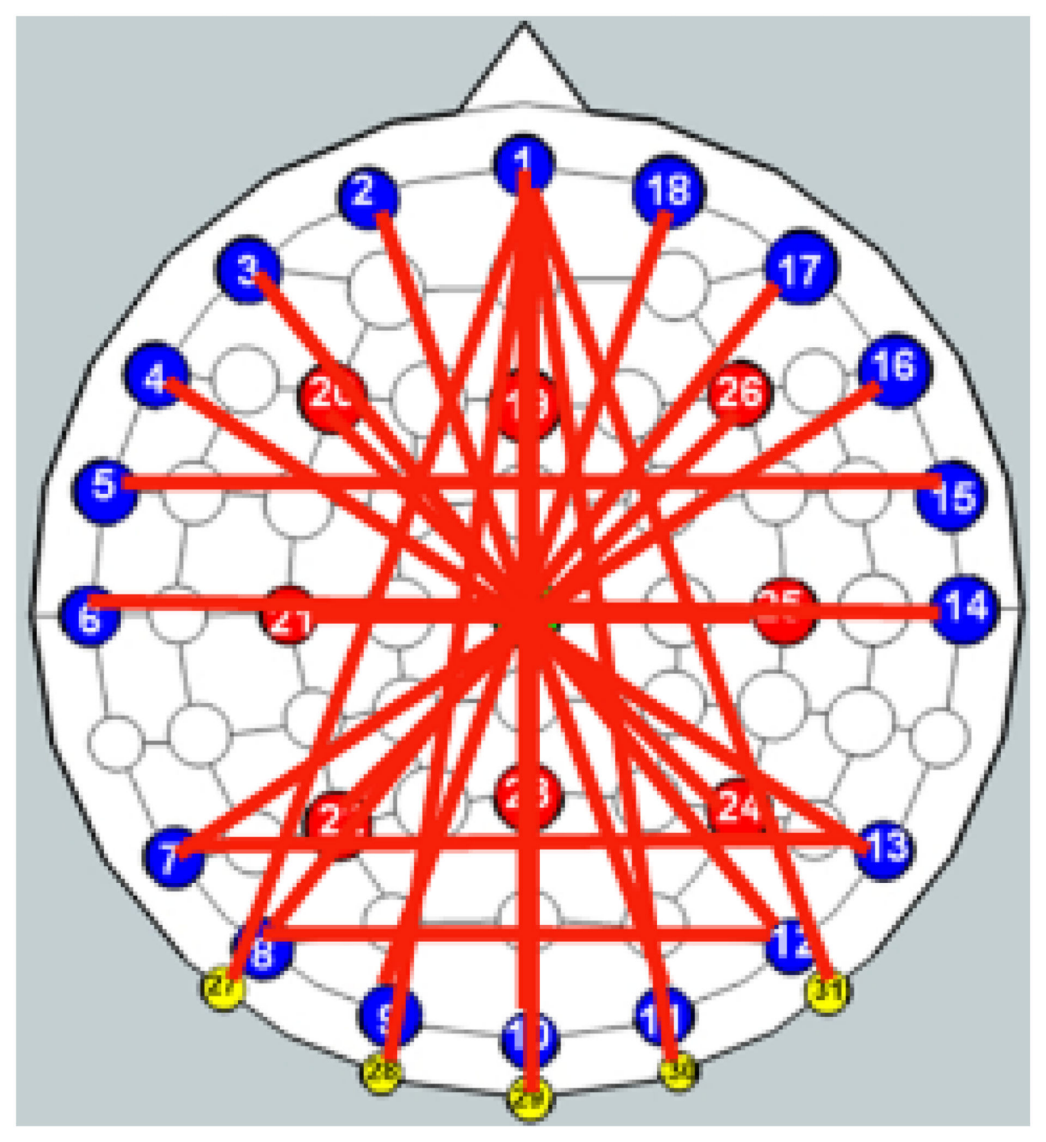

## I Introduction

MEDICAL application of Electrical Impedance Tomography (EIT) is well established, e.g. for imaging of lung function [1]–[4]. Application of EIT to the human head using scalp-mounted electrodes has been studied to pursue brain function imaging of evoked responses [5], during epileptic seizures [6] and for imaging of vascular trauma such as stroke [7]. Specifically for application to the human head using scalp-mounted electrodes in a tetrapolar measurement strategy, we have developed an EIT system to yield 100 frames per second (fps) called fEITER, for *functional Electrical Impedance Tomography of Evoked Responses,* first described in [8]. The system combines high frame rate and low measurement noise; its ability to measure cerebral hemodynamics is demonstrated here.

In the case of human head studies, convincing validation of EIT sensitivity to dynamic variation in cerebral perfusion status is an important step in its potential uptake for clinical use. However, such a demonstration is non-trivial in several respects, e.g. the wide variety of entirely normal phenomena that can influence the trans-impedance EIT measurement, and the safety of volunteer subjects. In developing the fast EIT system reported here, it was found that the trans-impedances recorded on the scalp undergo significant cyclical variation in synchronism with cardiac activity. This bio-impedance phenomenon, referred to as rheoencephalography (REG), has been widely studied in non-imaging mode [9], [10] and has shown the capability to track cerebral blood flow (CBF) [11]. Here, REG is explored further by EIT imaging in steady state.

Carotid artery occlusion is known to have a gross impact on cerebral perfusion [12]–[14]. Its transient application and the subsequent Transient Hyperemic Response (THR) is a well-established clinical procedure for assessment of cerebral autoregulation that can be performed safely by expert clinicians. The THR procedure is an excellent candidate for demonstration of EIT sensitivity to changes in cerebral hemodynamics. Clinical THR is usually monitored in real-time by Transcranial Doppler ultrasound (TCD) via the transtemporal acoustic window, which is sensitive to the blood flow velocity (FV) in the region of the middle cerebral artery (MCA), thus providing additional information for use in EIT validation. During long-term (60 s) carotid artery occlusion on human volunteers, Shi *et al.* [15] have previously reported 2-dimensional EIT imaging using a system with a single plane of 16 electrodes. We present here 3-dimensional EIT imaging during and after carotid artery occlusion, as a means to validate EIT sensitivity to cerebral hemodynamics.

## II Methods

### A fEITER Mk.I

The EIT measurements in this paper were made using two variants of our fEITER instrument. The original version of the system (as used in [8]) is termed fEITER Mk. I. It comprises an EIT subsystem which connects to a control/data storage laptop. Within the EIT sub-system’s Headbox unit, analogue and digital circuits for EIT are implemented on an 8-layer PCB that also houses a Xilinx Virtex-4 SX35 FPGA. The Headbox is connected to the human subject via snap-on, touch-proof non-shielded cables (Integral Process S.A.), 0.6m long, to low-impedance electrodes (Zipprep™, Covidien Inc.) placed on the scalp [16].

High-purity sinusoidal current injection (10 kHz, 1 mA_pk–pk_) is applied via any two selected electrodes by a pair of mirror-image Voltage-Controlled Current Sources (VCCSs) based on an enhanced Howland design [17]. The choice of 10 kHz is motivated by instrumental performance rather than physiological considerations. Low frequencies, just above the 1/f noise corner, offer high CMRR and, hence, measurement accuracy. Higher probe frequencies, however, permit higher frame rates, reducing the change occurring within a dynamic subject during a frame. We believe that measurement at 10 kHz with frame duration 10 ms (a small fraction of the cardiovascular period) is an appropriate compromise for parallel measurement EIT instruments, in the absence of a specific physiological requirement. The voltages between all numerically adjacent (non-injecting) electrode pairs are sampled simultaneously using parallel channels in which each channel has an instrumentation amplifier (AD8221, Analog Devices) and a 16-bit 500 kSps ADC (AD7686, Analog Devices).

**Fig. 1:**
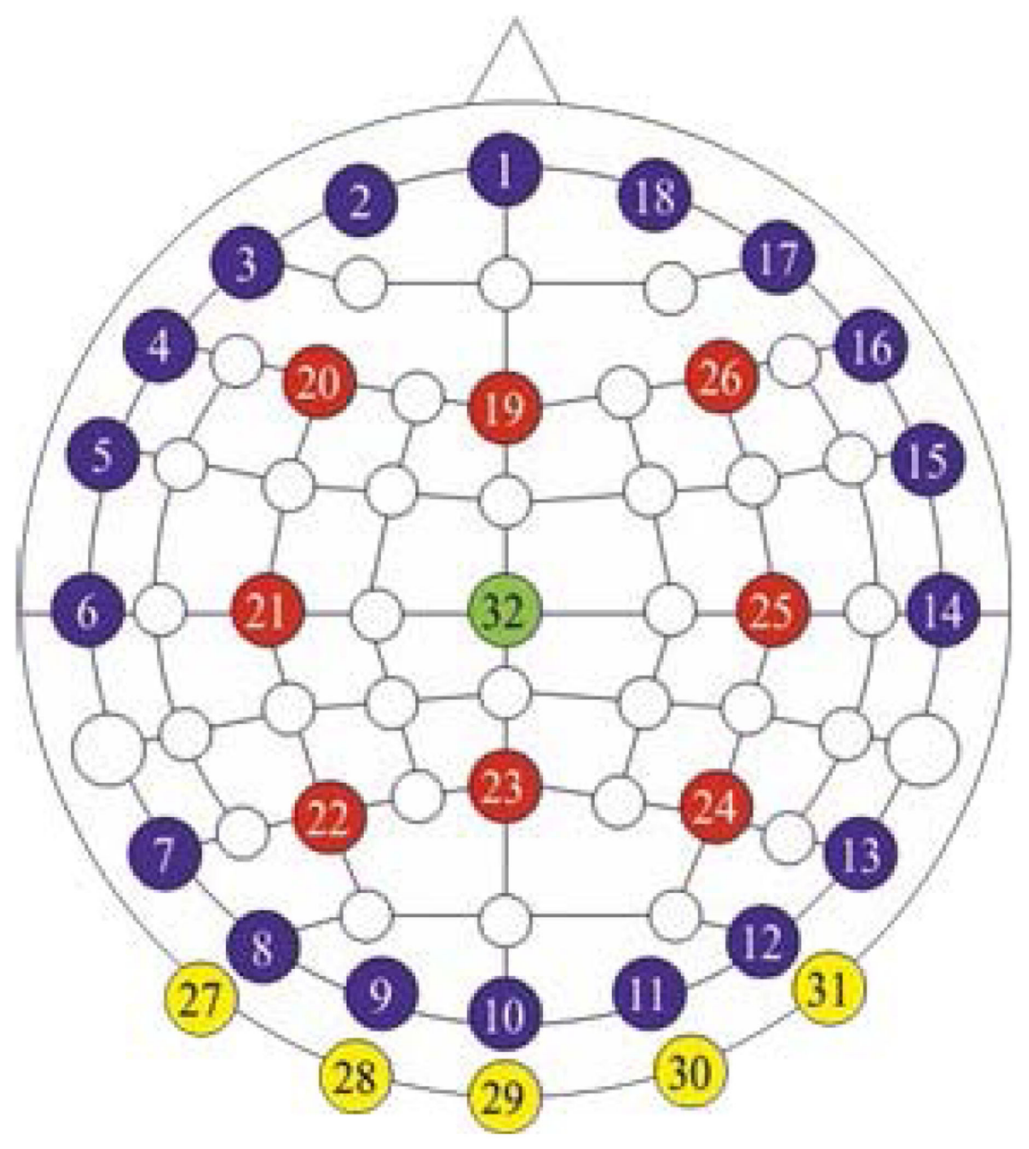
The fEITER electrode array (head anterior is at the top).

The 32 electrodes are placed at locations on the scalp based on the international EEG 10–20 system (Fig. 1), with an additional reference electrode placed on the lower mastoid region. Using CMOS switches, current injection electrode pairs are firmware-selected to be nearly diametrically opposed on the scalp. This injection protocol is chosen for maximal sensitivity in the central region of the head [18], due to the high conductivity of the scalp relative to that of the skull. The use of near-diametric current injection but adjacent voltage measurement is only partially compliant with the recommendations of studies such as [18], which advocate interface pattern angles between 90 and 180 degrees in 2D (planar) EIT geometries. The applicability of such guidance to the near-hemispherical problem treated here is uncertain, particularly as a significant proportion of fEITER electrode combinations are not coplanar. It should, nevertheless, be recognised that the measurement patterns used here are unlikely to be optimal.

Each selected pair provides a current pattern (CP) for 500 *μ*s whilst simultaneous voltage measurements are made by synchronous in-phase and quadrature demodulation using 200 samples from four cycles of the applied current (the first having been discarded), yielding a noise-equivalent bandwidth of 1.34 kHz. A sense resistor and identical voltage measurement to that described above measures the injected current, compensating for loading effects and providing patient safety protection. Twenty CPs are applied (see Table I) for each EIT frame, yielding 100 fps; each frame comprises 546 independent voltage measurements. To further enable low-noise trans-impedance measurement, each VCCS drives continuously a single, shielded, 'current bus' within the PCB, and CP switching occurs at zero values of the current waveform.

The Headbox is connected to the system laptop computer via a Base Unit that also provides power to all sub-systems, enables synchronization with auxiliary sensors (e.g. TCD), and acquires and formats data for export to the laptop. Using a local CPLD (Xilinx XC95144), the base unit carries out serial-to-parallel conversion of incoming data from the Headbox which are then acquired at 500 kS/s by a National Instruments USB DAQCard-6221 controlled using LabVIEW.

A static resistor network “phantom” was built to create load- and trans-impedances typical of those encountered in scalp-based EIT. The phantom was connected directly to the Headbox I/O terminals, eliminating connecting leads. When applied to the resistor network in this manner for measurement epochs of 50 s, i.e. 5,000 frames, fEITER Mk. I yields voltage measurement precision of 1061 nV_rms_ (averaged over all 546 measurements) and the result is almost identical for short measurement epochs of 0.5 s, i.e. 50 frames. The potential precision of this system is degraded by truncation error in the 16-bit readout of data that are 38-bits wide in the FPGA. With full-scale voltage measurement range 108.0 mV_rms_, the instrumental signal-to-noise ratio (SNR) limit of the system is 100.2 dB. When applied via connecting leads to representative tank geometries (i.e. introducing electrode effects), in various laboratory and hospital patient environments, the observed voltage measurement precision is in the range 3.1 to 4.2 *μ*V_rms_, depending on the environment. Individual channel SNRs in human trials were in the range 77 to 86 dB. The Mk I system has >90 dB CMRR at its 10 kHz measurement frequency. Hardware channel-to-channel gain matching is better than 2%, with software correction (based on calibration measurements of a common input, relative to a calibrated 6.5 digit voltmeter) applied to achieve CMRR-limited matching of 0.005% and an accuracy of 0.1%. The THR tests described in III.C below were carried out with fEITER Mk. I.

### B fEITER Mk.II

The original EIT subsystem has undergone some hardware and firmware developments, including:

(i) 24-bit voltage data readout, reducing the truncation error in the output data to 23 nV_rms_;

(ii) doubling the injected current amplitude to 2 mA_pk–pk_, still less than the patient auxiliary current limit of 1 m_Arms_ at 10 kHz (BS EN 60601-1:2006+A12:2014). This necessitated a small reduction (28%) in voltage gain, yielding full-scale voltage measurement range 151.0 mV_rms_.

The combined impact of these two changes is to enhance the instrument sensitivity, with signal amplitudes doubled and average voltage measurement precision of 803 nV_rms_ in the resistor phantom tests. Hence, the instrumental SNR limit of fEITER Mk. II is 105.5 dB. Due to the reduced truncation error, repeated voltage measurements form a smooth Gaussian distribution, with no visible evidence of quantization noise. Relative to the baseline voltages observed in human volunteer trials, the above precision yields SNR in the range 86-95 dB across all measurement channels. Hardware channel-to-channel gain matching is improved to 0.2% but CMRR and accuracy are identical to Mk. I.

### C Forward Modelling and Tissue Conductivities

The geometry of the head is complex, with five different tissue types (scalp, skull, cerebrospinal fluid (CSF), grey matter, and white matter), each having different conductivities. Therefore, a high-quality model of the head is required for forward calculations, obtained as follows.

From the geometric model of the tissue types, the measurement voltages *V* can be predicted from the discretization model function *U* of the conductivity distribution, with the modelling error *ε* and the measurement error *ε_m_* as in (1). If the model is accurate, *ε* is negligible; and *ε_m_* is small if measurement is carried out by a low-noise system. Employing Taylor expansion, *V* is approximated in a linear fashion by (2), where *J* is the Jacobian (‘sensitivity’) matrix and *i* is an iteration index. The estimated conductivity distribution $\hat{\sigma }$ can be computed by (3), using the iterative, regularised Gauss-Newton method as shown in (4) where *R* is a regularisation function and *λ* is the regularisation parameter [19]. The method based on (3) is often called the “absolute” method.

(1) $$V=U\left(\sigma \right)+\varepsilon \left(\sigma \right)+e_{m}$$

(2) $$V_{i}\approx U\left(\sigma_{i-1}\right)+J\left(\sigma_{i}-\sigma_{i-1}\right);\;where\;J=\frac{\partial U\left(\sigma_{i-1}\right)}{\partial \sigma }$$

(3) $${\hat{\sigma }}_{i}=\text{arg}\underset{\sigma }{\text{min}}\left\{{\left\|V_{i}-U_{\left(\sigma \right)}\right\|}^{2}\right\}$$

(4) $$\hat{\sigma }=\sigma_{i-1}+{\left(J^{T}J+\lambda R^{T}R\right)}^{-1}J^{T}\left(V_{i}-U\left(\sigma_{i-1}\right)\right)$$

*J* can be calculated from the integrals of the gradient of the dot product of the current and measurement fields [20]. The nominal tissue conductivities *σ_N_* can be estimated in a similar way to (4), setting all voxels of the same tissue type to a single nominal (isotropic) conductivity value. However, the average sensitivity of the CSF and the brain regions is very low relative to those of the scalp and skull (by factors of ~ 40 and ~ 1000, respectively). Therefore, to estimate the nominal conductivities of scalp and skull, the conductivities of the CSF, the grey and the white matter were fixed at constant values: 1.802 S/m for CSF [21] and 0.2849 S/m and 0.2556 S/m for the grey and white matter respectively [22]. Equation (4) can be re-written as (5), where ${\hat{\sigma }}_{sc-sk}$ is the vector of the scalp conductivity and the skull conductivity, and *J_sc–sk_* is the matrix of the average sensitivity of the scalp and the skull regions. The quality of the estimates can be evaluated using the Relative Error (RE) given by (6).

(5) $$\begin{array}{l} {\hat{\sigma }}_{sc-sk,i}=\sigma_{sc-sk,i-1}+{\left(J_{sc-sk}^{T}J_{sc-sk}+\lambda R^{T}R\right)}^{-1}\\ \;\times J_{sc-sk}^{T}\left(V_{i}-U\left(\sigma_{i-1}\right)\right) \end{array}$$

(6) $$Relative\;Error=\frac{\left\|V-U\left(\sigma \right)\right\|}{\left\|V\right\|}$$

The estimation process used 11 seconds of data (1,100 frames) recorded from a volunteer subject with the original fEITER system, on each of three separate days, all of the data being used to create a single average frame. The EIDORS forward modelling software [23] was used in conjunction with a geometric subject model composed of 337,744 elements (including the electrodes) constructed from a Magnetic Resonance image of the subject. Stable estimates were obtained after 15 iterations of (5), yielding average conductivity of the scalp 0.584 ± 0.026 S/m and of the skull 0.0084 ± 0.0005 S/m.

To test the prediction accuracy of these nominal conductivities, three further sets of fEITER measurement data from the same subject were averaged over 40 seconds (i.e. 4,000 records) each, and recorded on three separate days. This yielded RE = 0.32. The effect of using the same subject model, but without inclusion of the electrodes, was to increase RE by 0.04. These conductivity values were used in conjunction with several other non-subject models, with numbers of geometrical mesh elements approx. 53k, 158k, 208k, 274k (all based on the 53k model kindly provided by the UCL group of Prof. D. Holder [24]), and 301k. Only the first of these did not include the electrode geometry. The first and last of them performed significantly worse than the subject model, and the others performed almost as well as the subject model, the highest RE value being 0.37. For subsequent image reconstruction on all volunteer data reported here the 208k non-subject model was used, with assumed reference condition *σ_N_* values summarized in Table II, as reported in [25].

### D Image Reconstruction

The 546 voltage measurements collected by the fEITER system for each frame are from numerically adjacent electrode pairs. Of those, 37 do not correspond to spatially neighboring electrodes and are rejected from the image reconstruction process, leaving 509 independent voltage measurements. The skull conductivity was not varied in the reconstruction process, being fixed at the value in Table II. The electrode geometry was implemented manually.

The method of difference imaging is used here: a reference frame of measured data is selected; then the difference in the conductivity distribution between the reference frame and subsequent data frames is reconstructed from the voltage difference vector SV. Many authors, such as [26], have used difference imaging methods based on the linearized method of (3) above, and we call this the “traditional” method in the text below.

Recently, the issue of modelling error in EIT has attracted attention, e.g. [27]. In this work, we have used a novel nonlinear method of difference imaging that also incorporates an improved approach to the handling of modelling error. Equation (1) can be re-written as in (7), where *V_i_* is the measurement voltage vector at time index *i*, and the conductivity distribution at that time is *σ_i_*.

(7) $$V_{i}=U\left(\sigma_{i}\right)+\varepsilon \left(\sigma_{i}\right)+e_{m}$$

Then the difference of measurement vectors from time index *i* to time index *i* + 1 can be written as in (8).

(8) $$\begin{array}{l} \delta V_{\left(i+1,i\right)}=U\left(\sigma_{i+1}\right)-U\left(\sigma_{i}\right)+\delta \varepsilon_{\left(i+1,i\right)}+\delta e_{m}{}_{\left(i+1,i\right)};\\ \;where\;\delta \varepsilon_{\left(i+1,i\right)}=\varepsilon_{i+1}-\varepsilon_{i}\;and\;\delta e_{m}{}_{\left(i+1,i\right)}=e_{mi+1}-e_{m\;i} \end{array}$$

Applying Taylor series expansion to (8) yields (9), where *e_L_* is the linearization error due to neglecting the high order Taylor series terms.

(9) $$\delta V_{\left(i+1,i\right)}=J\left(\sigma_{i}\right)\delta \sigma_{\left(i+1,i\right)}+e_{L}+\delta \varepsilon_{\left(i+1,i\right)}+\delta e_{m}{}_{\left(i+1,i\right)}$$

Instead of using *σ_i_*, the nominal value of conductivity, *σ_N_*, is used in this paper. The estimation of conductivity at time index *i* + *1*, *σ*_*i*+1_, is then denoted ${\tilde{\sigma }}_{i+1}.$ If *σ_N_* is close to *σ_i_*, the Jacobian *J*(*σ_N_*) is close to *J*(*σ_i_*) and also *δσ*_(*i*+1,*i*)_ is close to $\delta {\tilde{\sigma }}_{\left(i+1,N\right)}.$ Then we have:

(10) $$\begin{array}{l} J\;\left(\sigma_{i}\right)\;\delta \sigma_{\left(i+1,i\right)}\approx J\;(\sigma_{N})\;\delta {\tilde{\sigma }}_{\left(i+1,N\right)};\\ \;where\;\delta {\tilde{\sigma }}_{\left(i+1,N\right)}={\tilde{\sigma }}_{i+1}-\sigma_{N} \end{array}$$

The right hand side of (10) can be transformed backward to the difference of the discretisation model function of ${\tilde{\sigma }}_{i+1}$ and that of *σ_N_* with Jacobian error *e_J_*, as shown in (11).

(11) $$J\;(\sigma_{i})\;\delta \sigma_{\left(i+1,i\right)}=U\;({\tilde{\sigma }}_{i+1})-U\left(\sigma_{N}\right)+e_{J}$$

*e_J_* is the result of using *J* (*σ_N_*) rather than *J* (*σ_i_*). Equation (8) can then be estimated by (12) below.

(12) $$\begin{array}{l} \delta V_{\left(i+1,i\right)}=U\;({\tilde{\sigma }}_{i+1})-U\;(\sigma_{N})+\varepsilon_{\delta \left(i+1,i\right)}+\delta e_{m\left(i+1,i\right)};\\ \;where\;\varepsilon_{\delta \left(i+1,i\right)}=e_{J}+e_{L}+\delta \varepsilon_{\left(i+1,i\right)} \end{array}$$

Since *U*(*σ_N_*) is a known-constant vector as well as *δV*_(*i*+1,*i*)_, the new known-parameter ${\tilde{V}}_{\left(i+1,i\right)}$ (*σ_N_*) can be defined as ${\tilde{V}}_{\left(i+1,i\right)}(\sigma_{N})=U(\sigma_{N})+\delta V_{\left(i+1,i\right)},$ and then (12) can be rewritten in the compact form of (13).

(13) $${\tilde{V}}_{\left(i+1,i\right)}\left(\sigma_{N}\right)=U\left({\tilde{\sigma }}_{i+1}\right)+\varepsilon_{\delta \left(i+1,i\right)}+\delta e_{m\left(i+1,i\right)};$$

(14) $${\tilde{\sigma }}_{\left(i+1,i\right)}=\underset{\tilde{\sigma }}{\arg\min}\left\{{\left\|{\tilde{V}}_{\left(i+1,i\right)}\left(\sigma_{N}\right)-U\left(\tilde{\sigma }\right)\right\|}^{2}\right\}$$

(15) $$\delta {\hat{\sigma }}_{\left(i+1,i\right)}={\tilde{\sigma }}_{\left(i+1,i\right)}-\sigma_{N}$$

Noticeably, although (13) was based on the difference approach, it looks similar to the absolute approach of the forward model given in (1). The new modelling error term *ε*_*δ*(*i*+1,*i*)_ is practically impossible to estimate, and it essentially has to be ignored. Note that *ε*_*δ*(*i*+1,*i*)_ is expected to be substantially smaller than *ε*. The absolute image based on *σ_N_* can be computed by (14), and the desired difference image can be computed from (15), with regularisation to be applied separately.

The advantages of this method are that (a) it is nonlinear, and (b) the modelling error is substantially reduced to be *ε*_*δ*(*i*+1,*i*)_ which is smaller than *ε*_*i*+1_ or *ε_i_*. The reconstruction performance depends on the magnitude of the term *ε*_*δ*(*i*+1,*i*)_ ignored above, requiring *δσ*(_*i*+1,*i*)_ to be very small and *σ_N_* to be accurate. Note that the use of a generic model rather than a subject model impacts the magnitude of *ε*_*δ*(*i*+1,*i*)_ also. However, the error from this approach is still much smaller than that of the method based on (3) above.

With over 208,000 elements in the head model, and over 500 measurements per frame, image reconstruction for head EIT is a large-scale computational problem. In particular, the memory requirement for the term *J^T^ J* runs to many GBytes. To render the computation tractable on readily available desktop systems, the image reconstruction technique used was the Regularised Newton-Krylov GMRes method, with 200 Krylov subspaces. Briefly, the technique contains the following building blocks:

(i) For a large-scale problem in the form of (16), the Krylov projection method reduces the scale of the problem substantially. It was first applied to EIT data by Polydorides *et al.* [28] and was later developed [29] to use the Arnoldi implementation, as we have done in this work;

(16) Ax=b

where *b* is the observed data, *A* is the known transfer matrix, and *x* is the unknown variable;

(ii) For the reduced-scale problem, Novati and Russo [30] have shown that the popular Generalised Minimal Residual (GMRes) method is applicable, even in severely ill-conditioned cases;

(iii) The problem is now at a scale where conventional solution methods can be applied, e.g. the Gauss-Newton method;

(iv) Second-order iterative Tikhonov regularization [31] was applied, as shown in (17). The smoothness prior *R* was selected with *λ* = 1 × 10^−7^, which was evaluated by the L-Curve method [32] applied to human head data from one subject undergoing the THR test;

(17) $${\hat{x}}_{i+1}={\hat{x}}_{i}+{\left(A^{T}A+\lambda R^{T}R\right)}^{-1}A^{T}\left(b-A{\hat{x}}_{i}\right)$$

(v) This overall process is expressed as in (18), where *A* has become $\tilde{A},$ *b* has become $\delta \tilde{b}$ and *x* has become *δx*. This version of the problem can fit into the memory when the matrix multiplications are ordered properly in the projection process;

(18) $$\begin{array}{l} \tilde{A}\delta x=\delta \tilde{b};\;where\;\tilde{A}=\left(A^{T}A+\lambda R^{T}R\right),\\ \delta x=\left({\hat{x}}_{i+1}-{\hat{x}}_{i}\right),\;\delta \tilde{b}=A^{T}\left(b-A{\hat{x}}_{i}\right) \end{array}$$

(vi) In the iteration process, a new step-length strategy [33] was applied in order to avoid divergence. After application of the above image reconstruction technique to various simulations, experimental tank tests and human data, the number of iterations was set to 15.

**Fig. 2:**
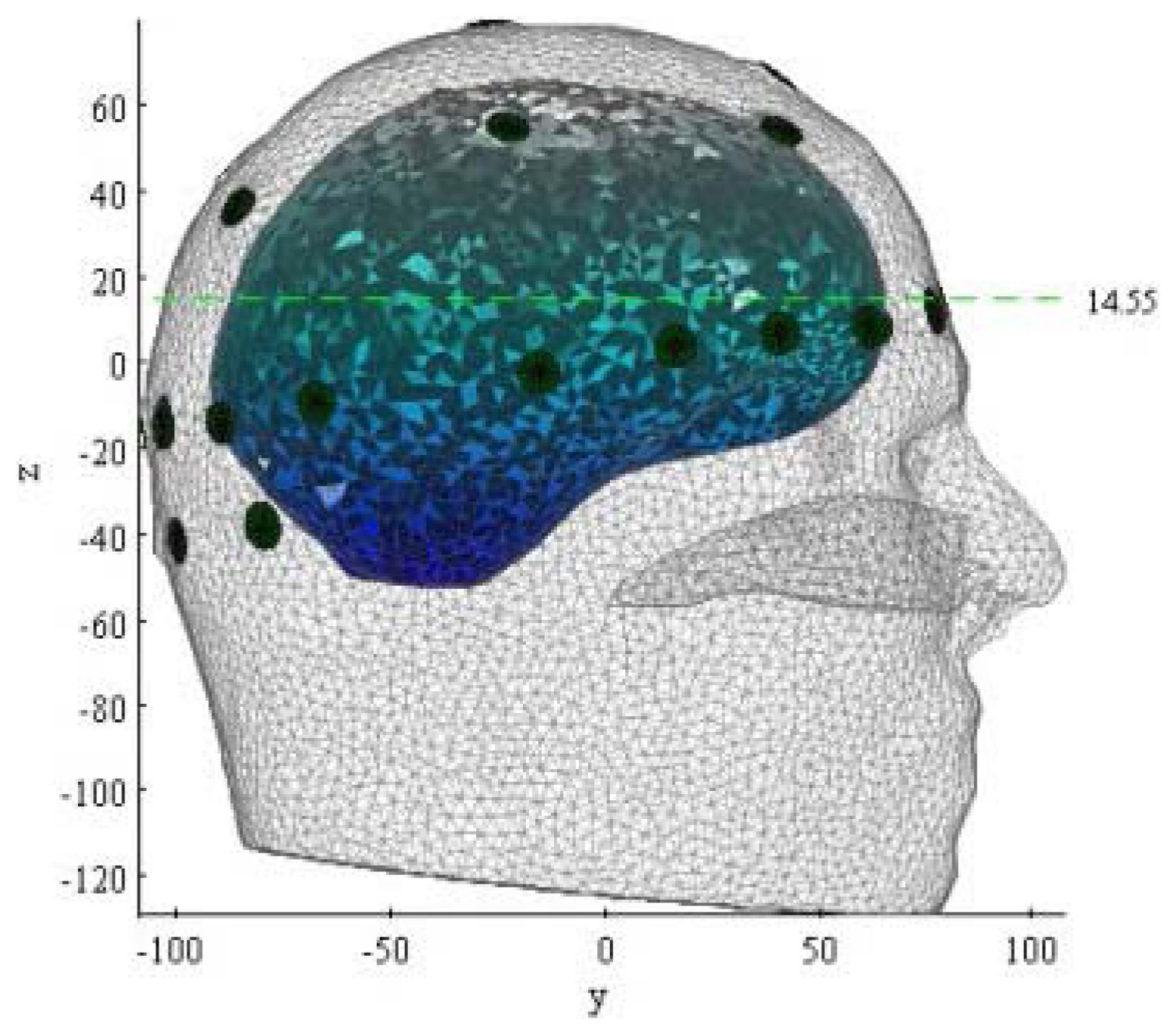
The green dashed line shows the position of the 2-dimensional cross-section used in Figs. 7, 8, 10 and 12 below. The dark circles show the electrodes visible from the right of the subject. The graded blue-grey area shows the white and grey matter (projected).

For human test data, the initial guess tissue conductivities were set to the values in Table II above. Under these conditions, the Jacobian matrix for reconstruction of human head test data was approx. 900 Mbytes and the necessary computation time for each frame of data was approx. 40 minutes. Throughout this paper, the 2-dimensional cross-section chosen to illustrate the reconstructed 3-D images from human tests is that shown in Fig. 2.

### E REG Recordings

As will be presented in sub-section III.B, the REG phenomenon is readily observable in resting, unstimulated subjects with both versions of fEITER, recording at 100 fps. More than 50 resting subjects were measured with fEITER Mk. I over a period of several years. In the case of Mk. II, three volunteer subjects from the research team were measured, and repeat measurements were carried out on one subject to investigate the reproducibility of the results, including measurements on different days (with application of new scalp electrodes).

In the discussion below, all voltages are root-mean-square (rms) values. Throughout this paper, each individual EIT measurement is labelled by its current injection and voltage measurement electrodes, e.g. the tetrapolar measurement 1-30-2-3 indicates the voltage measured between electrodes 2 and 3 for current injection between electrodes 1 and 30. An individual voltage measurement can be extracted from successive frames to yield a 100 Hz time-series, as in Figs. 5, 6, 9 and 11 below.

Image reconstruction by the above method of difference imaging was carried out as follows: (a) the reference frame of voltage measurement values was set to the average values for two frames at 60 ms before the rapid fall of EIT voltage amplitude, and (b) measurement data for subsequent pairs of frames were averaged, yielding temporal resolution of 50 fps.

### F THR Tests

The THR test is performed by compressing the common carotid artery in the neck for a few seconds. This leads to a predictable fall in blood flow to the brain during compression, shown as reduced FV in the MCA by TCD measurement, followed by a transient increase in FV above baseline when pressure is released [12], [13]. The THR tests reported here were carried out at the Salford Royal NHS Foundation Trust, and approval was obtained in advance from a National Health Service Research Ethics Committee (REC 11/YH/0346) and the Medicines and Healthcare Products Regulatory Agency (MHRA CI/2011/0045).

The THR process was carried out 17 times on five volunteer subjects from within the research team. In each THR test, a 5-second (approx.) occlusion was applied to the left or the right MCA whilst recordings were made of data from EIT (fEITER Mk. I), TCD (MultipDop T-series, ScanMed Medical) and ECG (Mindray PM8000E Monitor).

For EIT image reconstruction by the above method of difference imaging, the reference frame of voltage measurement values was set to the average values for a complete REG (i.e. cardiac) cycle immediately prior to the start of the THR occlusion.

## III Results

### A Imaging System Performance Assessment

Successful imaging within the human head using fEITER with scalp-mounted electrodes is critically dependent upon two attributes of the methodology described in Section II: low-noise measurement, as discussed in sub-sections II.A and II.B; and the capability of the software algorithm and modelling package to produce images as discussed in sub-section II.D by using a geometric head model that is not derived from anatomical scans of each human subject, i.e. a non-subject model as described in sub-section II.C. The performance of the methodology is assessed here by tests using both physical and computer-simulated phantoms.

#### 1) Experimental Phantom

A head-shaped tank was filled with a saline solution of conductivity 2.7 S/m for tests as described in [34], one of which is described here. Thirty-two electrodes (10 mm dia.) were placed on the tank wall, in direct contact with the saline solution, in the same geometry as described in Section II, and the same measurement protocol was implemented, using fEITER Mk. I. The measurements were averaged over a 0.5s period (50 records) to minimize measurement noise. A geometric model with 93,687 elements was created from a human MRI image, but with various dimensional differences compared with the tank, e.g. the nasion to the inion distance was 360mm for the tank, but it was 380mm for the human head (as used for the inverse model). Hence, the modeling error *ε* was large in this case.

A cylindrical piece of carrot (150 mm long and 27 mm in diameter, of conductivity 0.068 S/m) was inserted into the saline. With its long axis parallel to the vertical axis of the tank, it was placed to the left of center, then to the right, and then withdrawn. To track the movement of the carrot, difference imaging was applied, using a reference frame that was recorded prior to insertion. The traditional image reconstruction method (3) and the proposed new method (14) were applied, as described in sub-section II.D.

**Fig. 3:**
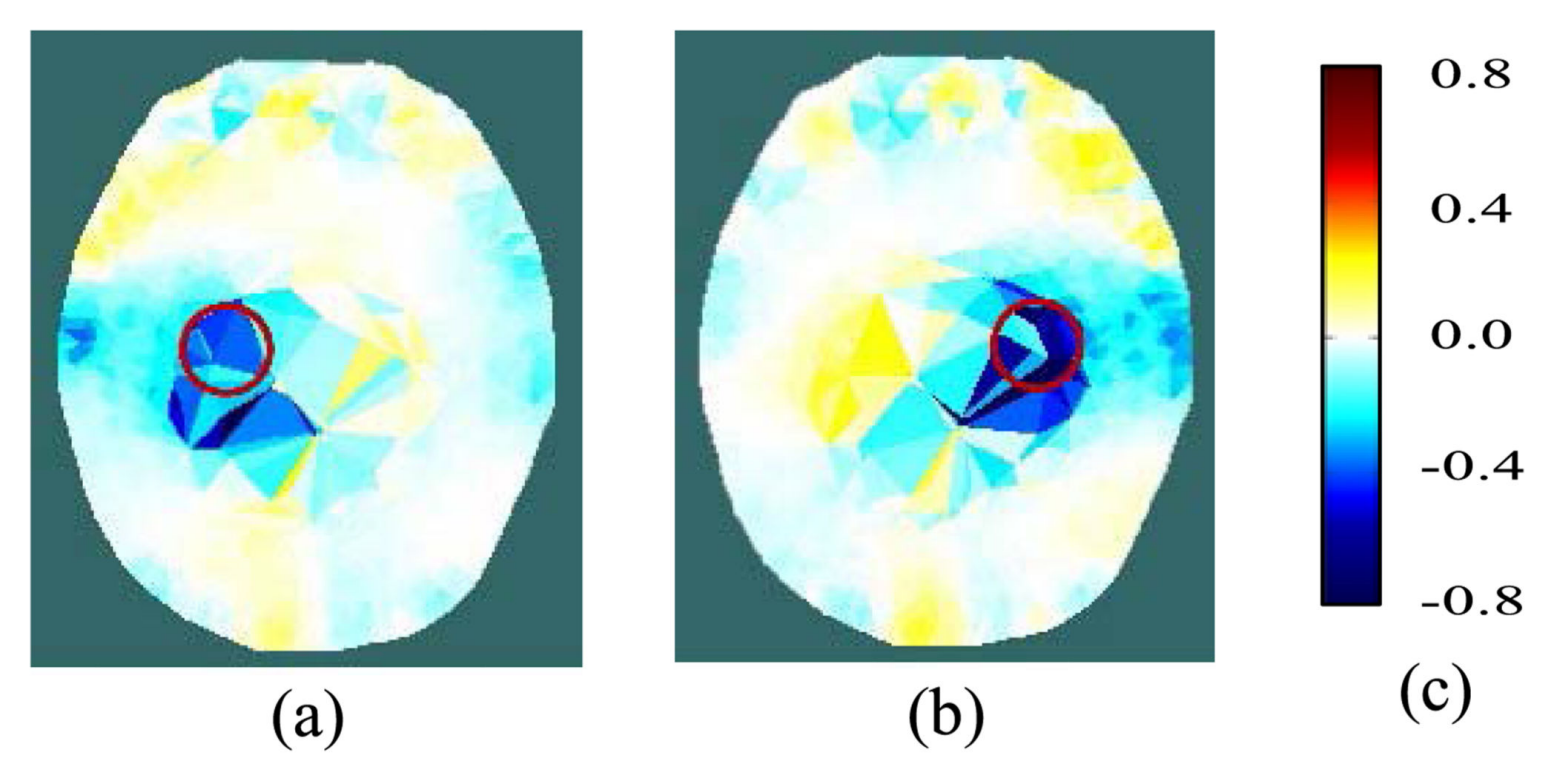
Reconstructed images of a carrot inclusion (shown by the red circle) in a saline solution within a head-shaped tank: (a) carrot in left-of-center region; (b) carrot in right-of-center region; (c) color legend of conductivity difference (S/m).

The traditional method failed to produce images that resembled the physical distribution in any respect. The proposed new method yielded images that successfully localized the carrot and its movement, as illustrated in Fig. 3. The position of the carrot is resolved, despite the modelling error and the coarse resolution of the data inversion FEM in this region, although both contribute to inaccurate estimation of the conductivity change.

#### 2) Simulation

**Fig. 4:**
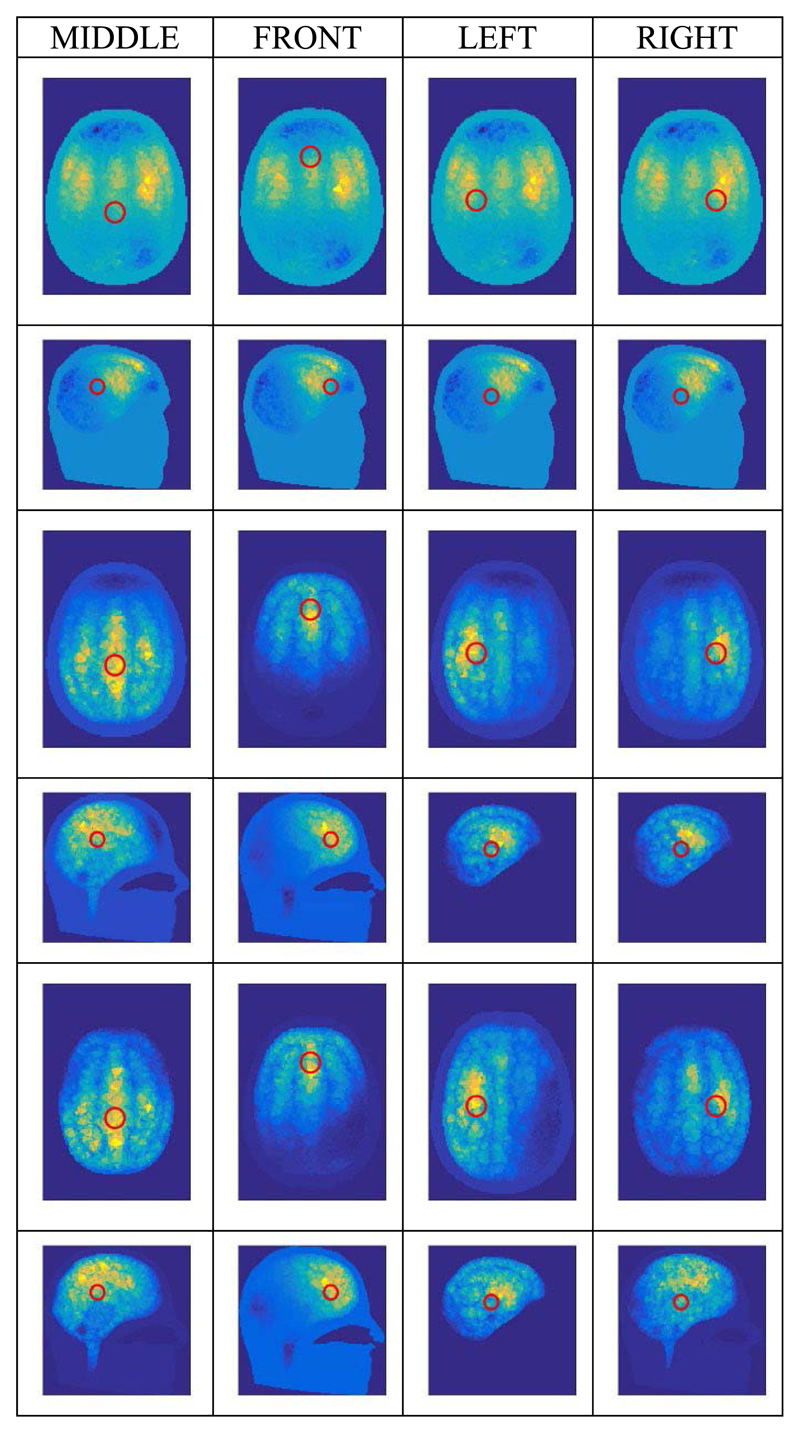
Reconstructed images (axial and sagittal views, sectioned at the CoG) of conductivity change due to introducing the “blood” sphere in four different locations indicated by the red circles (showing the projection of the sphere onto the CoG cut planes): top 2 rows, using the traditional method, with no noise; middle 2 rows, using the new method, with no noise; bottom 2 rows, using the new method, with 70 dB SNR. The location of the nose is at the top in the axial images, and on the right for the sagittal images. The numerical range associated with the color scale of conductivity change is varied between simulations to best illustrate the localization performance in each case, but it is fixed within each pair of views (axial and sagittal).

The system performance was assessed by simulating the inclusion of a 20 mm-diameter sphere of conductivity 0.65 S/m (i.e. blood) at four different locations inside the head, as shown in Fig. 4. The UCL-274k element model (see sub-section II.C) was used as the forward model and the UCL-208k element model as the inverse model, as chosen in sub-section II.C for all human head cases. Hence, the modelling error in this case is moderate. Apart from the inclusions, only three tissue conductivities were used in these simulations: the scalp, skull and white matter values were set as in Table II; the conductivities of the grey matter and the CSF were set to that of the white matter.

In the simulations, the current patterns listed in Table I were applied and 509 adjacent (simulated) measurements were obtained. Simulations were performed for three different measurement conditions: no noise (i.e. SNR is ∞); 70dB SNR (repeated 10 times); and 50dB SNR (repeated 10 times). For each reconstructed image, the location of the so-called center of gravity (CoG) was calculated in accordance with [35], in order to estimate the localization error. For the CoG calculation, the tetrahedral inverse model was converted into a 2.04M-voxel model with resolution of 1.5mm. For the presentation here, the conductivity change of the scalp was removed from the reconstructed images in order to display only the change within the cranium region.

The images (axial and sagittal views) resulting from the traditional reconstruction method of (3) are shown in the top two rows of Fig. 4 for the no-noise case; they are all almost identical to each other, irrespective of the position of the inclusion. Hence, the localization is considered to have failed. The same behavior is observed for the traditional method in the 70 dB and 50 dB SNR cases (not shown here).

Fig. 4 also shows the images resulting from the new proposed reconstruction method of (14), for both the no-noise and 70 dB SNR cases. The distance from the center of the original inclusion to the CoG of each reconstructed image is given in Table III. From visual inspection of Fig. 4 and from consideration of the CoG values, the new method of (14) is seen to achieve good localization of all four inclusions and they are all well distinguished from each other. The images for the 70 dB SNR case are very similar to those from the no-noise case, both visually and in terms of CoG values. At 50 dB SNR however, even using the method of (14), Table III shows that the inclusion-to-CoG distances are relatively large, with a much greater scatter of values between repeats.

Many other simulations were performed with the above inclusions and with different combinations of forward and inverse models that present larger modelling errors than the case discussed above. In all cases, the proposed new method of (14) achieved good reconstructions, whilst the traditional method of (3) failed to achieve any acceptable reconstructions.

### B REG

**Fig. 5:**
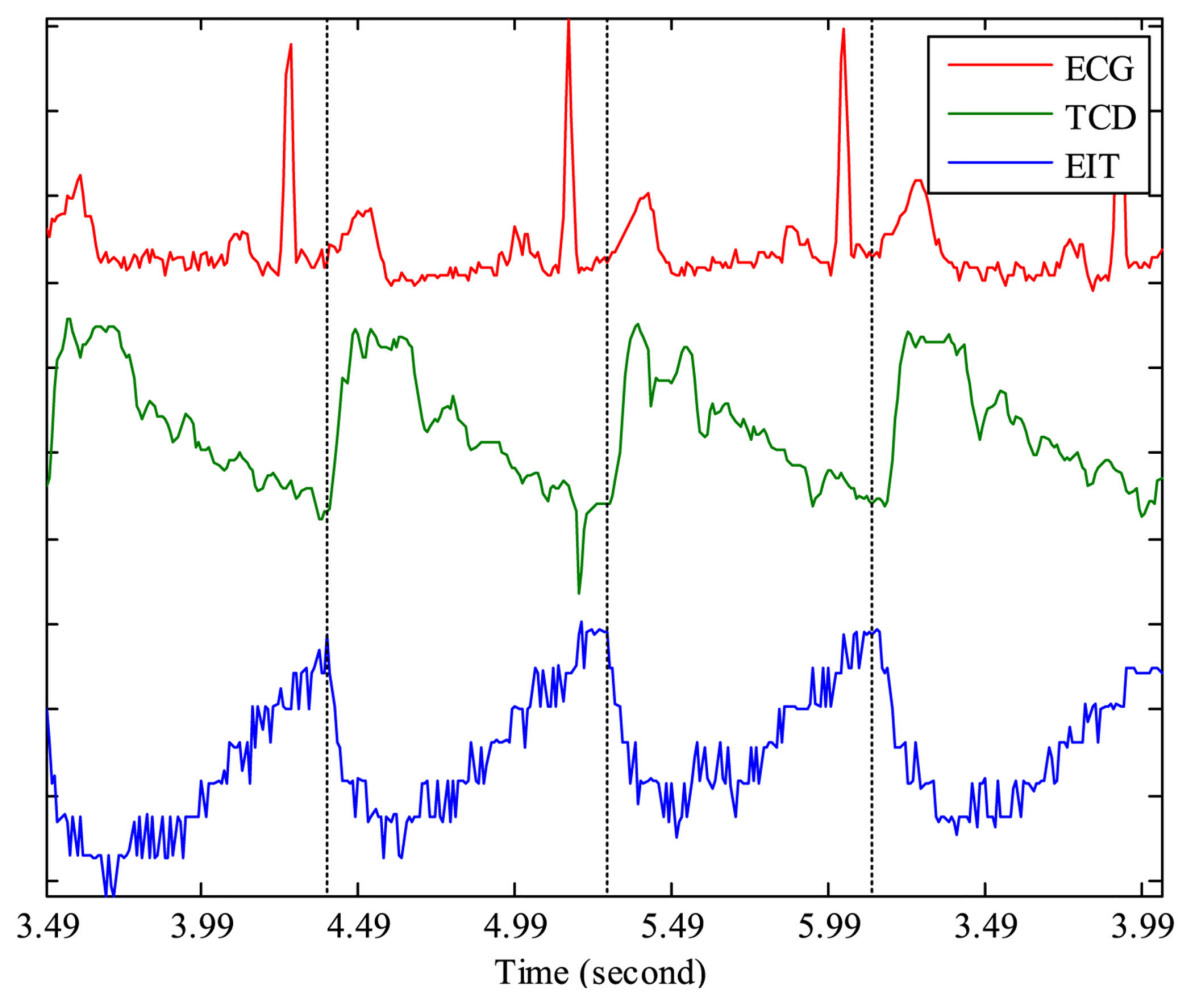
Examples of the time course of simultaneous recordings from ECG, TCD and fEITER Mk. I systems (measurement 1-10-2-3).

Fig. 5 shows typical traces from the ECG, TCD and fEITER Mk. I systems taken during THR tests, in the period before application of the carotid clamp. The TCD data monitor blood FV in the MCA.

The raw EIT signals in Fig. 5 show the REG cycle, which is synchronous with the ECG signal, the lowest EIT voltage occurring in this case typically 150 ms after the R-wave peak in the ECG, in line with expected pulse delay [36]. The EIT data in Fig. 5 are typical of recordings made in all subjects with Mk. I; REG is always present, but is not resolvable in all tetrapolar measurements, due to the quantization noise floor. The rapid fall in the magnitude of the EIT signal always corresponds closely with the rapid increase in the magnitude of the TCD signal. This supports the attribution of these EIT signal variations to the REG process whereby the pulsatile flow of blood into the head results in localized changes in conductivity.

**Fig. 6:**
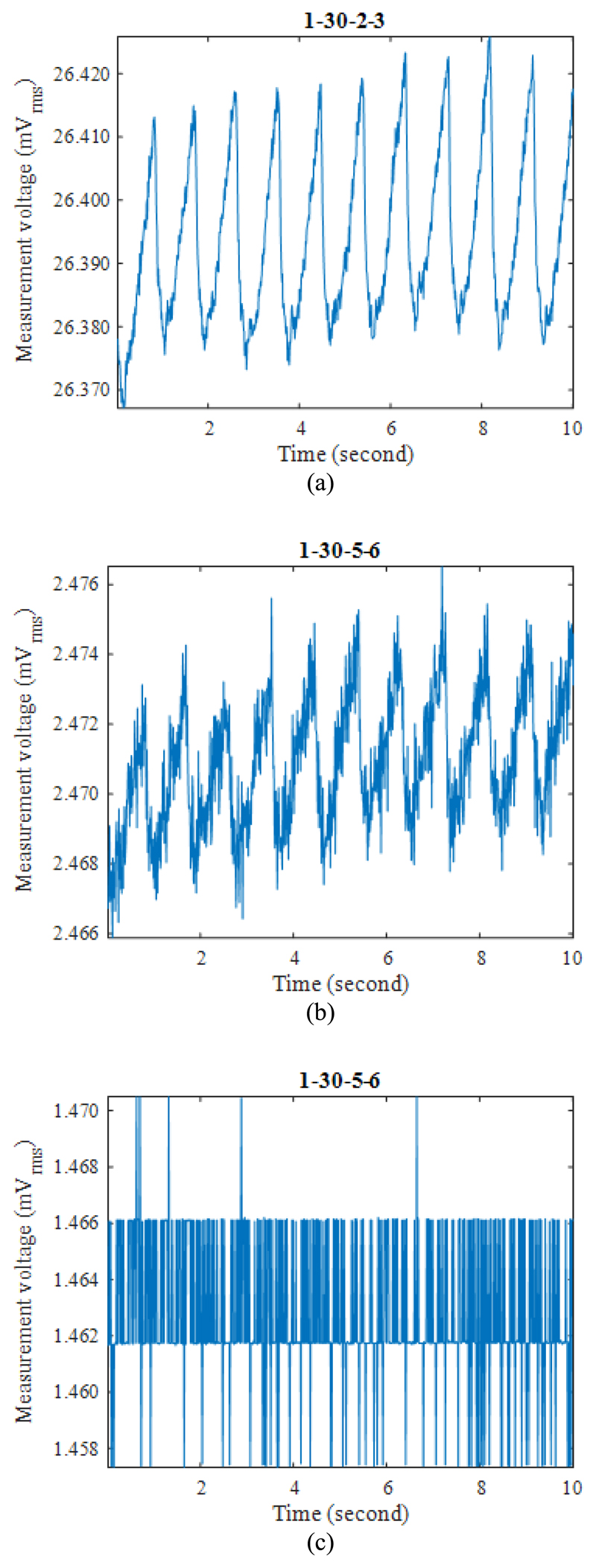
Examples of EIT measurements on a resting subject (electrode configurations indicated): (a) and (b) with fEITER Mk. II; and (c) same configuration as in (b), but with fEITER Mk. I (on a different subject).

Fig. 6 (a)-(b) show examples of improved measurements taken with fEITER Mk. II, displaying excellent resolution of REG signals. For voltage measurement sites close to the current injection electrodes, as in Fig. 6(a), the variation attributed to REG can be up to approx. 50 *μV.*

The use of the Mk. II system allows measurement of REG in lower trans-impedance situations (Fig. 6(b)) that would have been obscured by quantization noise using the Mk. I (Fig. 6(c)). The observed oversampling of the REG waveform suggests 5-10 dB of SNR improvement is available through bandwidth reduction. This applies equally to the Mk. I and Mk. II instruments.

**Fig. 7:**
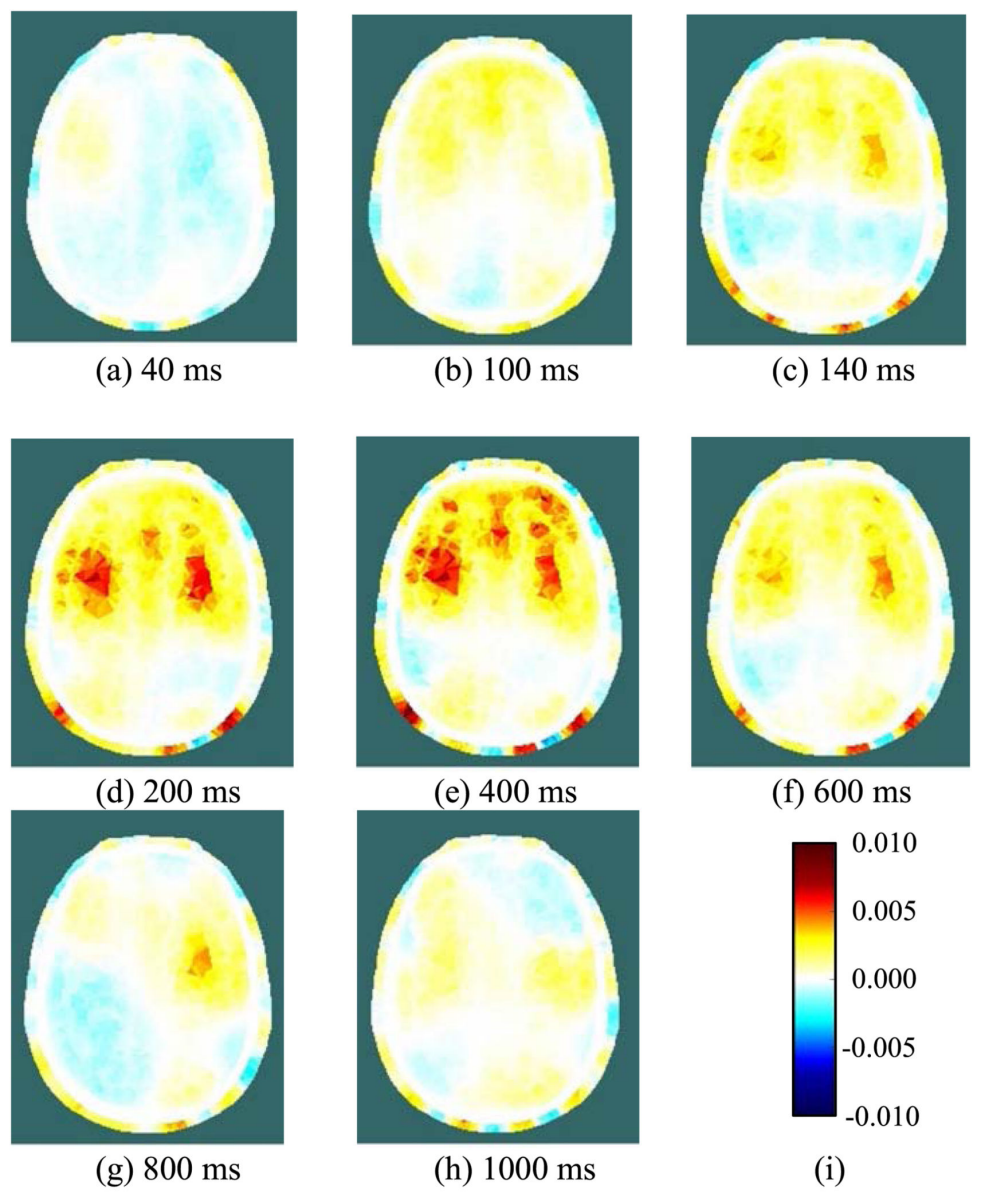
(a)-(h) Reconstructed images of conductivity change during a single REG cycle, at the indicated times after the reference period; (i) legend for all images, depicting the change in conductivity (units S/m).

**Fig. 8:**
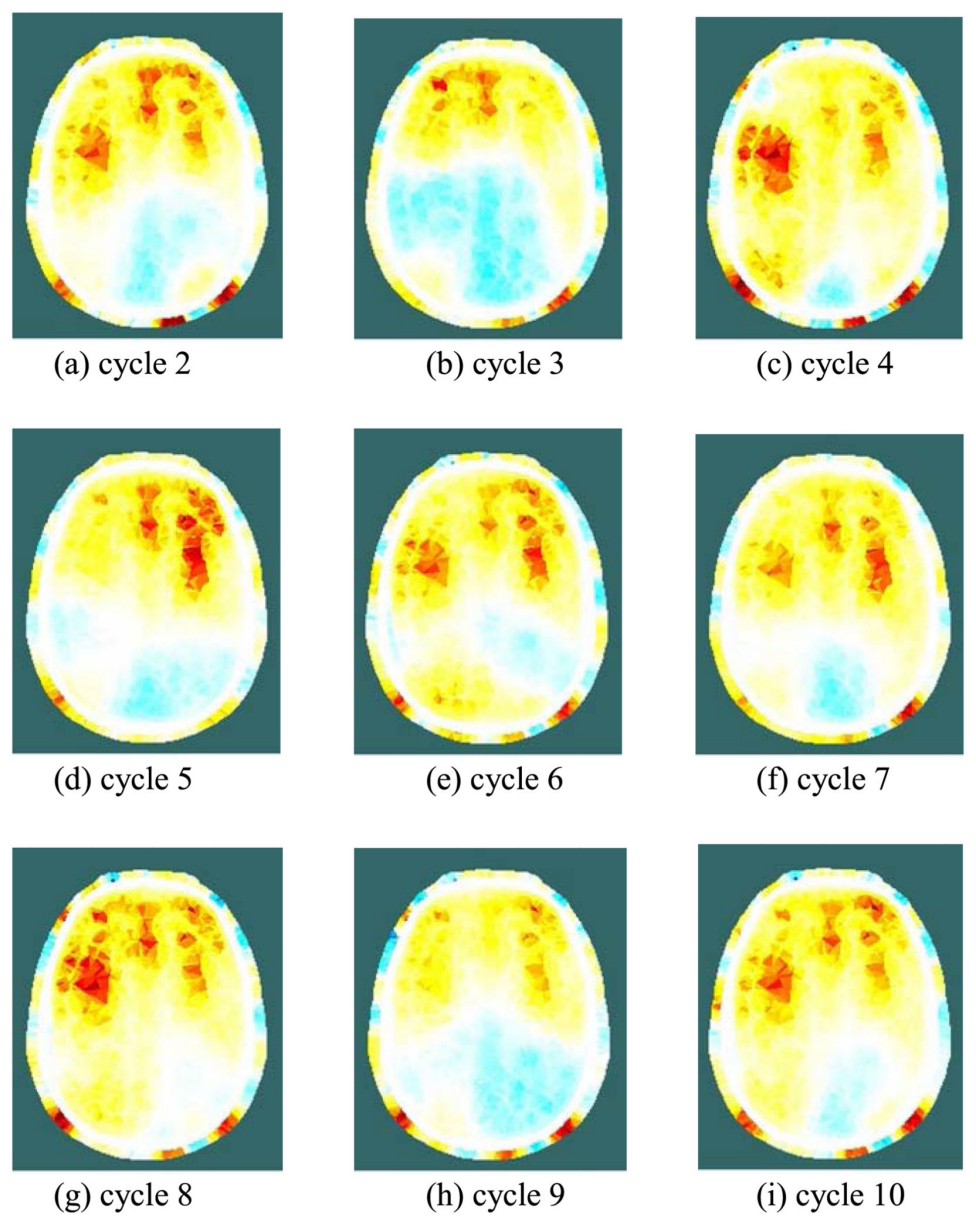
Reconstructed images of conductivity change during the REG cycle, at 200 ms post-reference, for 9 consecutive cycles immediately after the cycle shown in Fig. 7 (cycle 1). The legend is shown in Fig. 7(i).

Figs. 7 and 8 show, for one of the three subjects, examples of the conductivity change during REG. Specifically, they show the 2-D section, as illustrated in Fig. 2, through the reconstructed 3-D images of conductivity change, relative to the reference period. Fig. 7(a)-(h) shows the temporal development of conductivity change at eight selected times during a single REG cycle. At 40 ms after the reference period, Fig. 7(a) shows only very slight, and perhaps not significant, changes from the reference condition. In Fig. 7(b), at 100 ms after the reference period, a systematic increase of conductivity emerges in the forward area of the plane, which is then resolved into three areas of high conductivity increase over the following 100 ms (Fig. 7(c)-(d)). These areas of high conductivity increase persist for several hundred ms (Fig. 7(e)-(f)) before they gradually fade away (Fig. 7(g)-(h)), prior to the next REG cycle.

Fig. 8 shows, for each of nine consecutive REG cycles immediately after the cycle shown in Fig. 7, the reconstructed image at 200 ms after the reference period. Although significant cycle-to-cycle variation can be seen, the locations and magnitudes of the conductivity changes are highly correlated between cycles. The images of Figs. 7 and 8 show significant conductivity change in the scalp as well as the grey and white matter.

During the REG cycle, the peak local magnitude of the conductivity change of the grey/white matter (relative to the reference period) within the 3-dimensional images, is of the order of 1%. Data taken on different days with the above subject were found to be highly repeatable and reconstructed images show clearly the same general behaviour. For each of the other two subjects measured with Mk. II, only a single trial session was conducted; in each case, the measurement data showed that several electrodes were poorly attached (e.g. showing increased signal noise and/or reduced REG voltage variation), thus reducing the quality of the reconstructed images. Nevertheless, for the many well-attached electrodes, similar behavior of EIT voltages, as described for the first subject above, was observed during the REG cycle.

### C THR

**Fig. 9:**
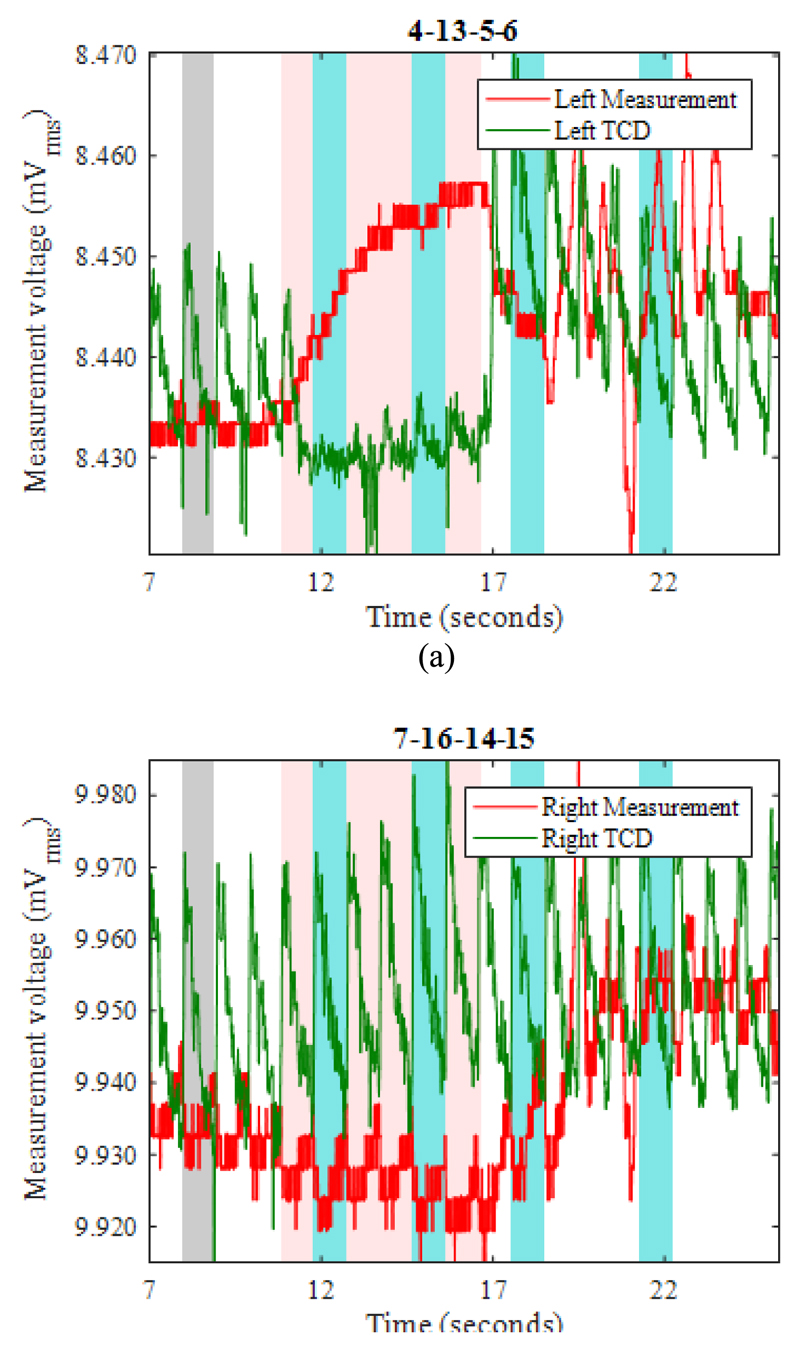
For LEFT carotid artery occlusion during the period 11.0-16.8s (shaded pink), EIT measurements (red) and TCD measurements (green), recorded near (*a*) the left ear, and (*b*) the right ear. Grey shading shows the reference period for EIT image reconstruction.

Fig. 9(a)-(b) show example EIT data and TCD data before, during, and after occlusion (pink shading) of the left carotid artery. The blood flow velocity on the occluded side (Fig. 9(a)) is temporarily reduced, as shown by the near-disappearance of the minimum-to-maximum variation in the TCD signal (denoted “min-max amplitude” here). Once the occlusion is relieved, the TCD signal shows much-enhanced variation compared with the pre-occlusion period, before it settles down again within about 5s. During the occlusion, the TCD signal on the non-occluded side shows a slightly increased min-max amplitude, suggesting enhanced cerebral blood flow. Similar features are observed in the great majority of the THR trials, as can be seen for the right-side occlusion on the same volunteer in Fig. 11(a)-(b). In fact, the TCD data in Fig. 11(b) continue to show significant blood flow velocity during the occlusion, albeit with reduced min-max amplitude, suggesting that the occlusion was not fully effective in this particular trial.

During the left-side occlusion, the EIT measurements near the left ear (Fig. 9(a)), show a substantial increase in the measurement baseline, followed by large fluctuations after the occlusion is relieved. The EIT data in Fig. 9(b) for the geometrically opposite measurement near the right ear, i.e. on the non-occluded side, show a small fall in baseline during the occlusion, followed by significant variations after the occlusion is relieved. Similar trends are seen in Fig. 11(a)-(b) with respect to the right-side occlusion. These trends in the EIT measurements near the ears are seen clearly for 15 out of 17 THR trials. It is worthwhile to emphasise that the 3-dimensional image reconstruction process uses all 509 nearest-neighbor measurements for each frame, whereas Figs. 9(a)-(b) and 11(a)-(b) show only two examples of those measurements (per frame), to illustrate the behavior of the raw data.

**Fig. 10:**
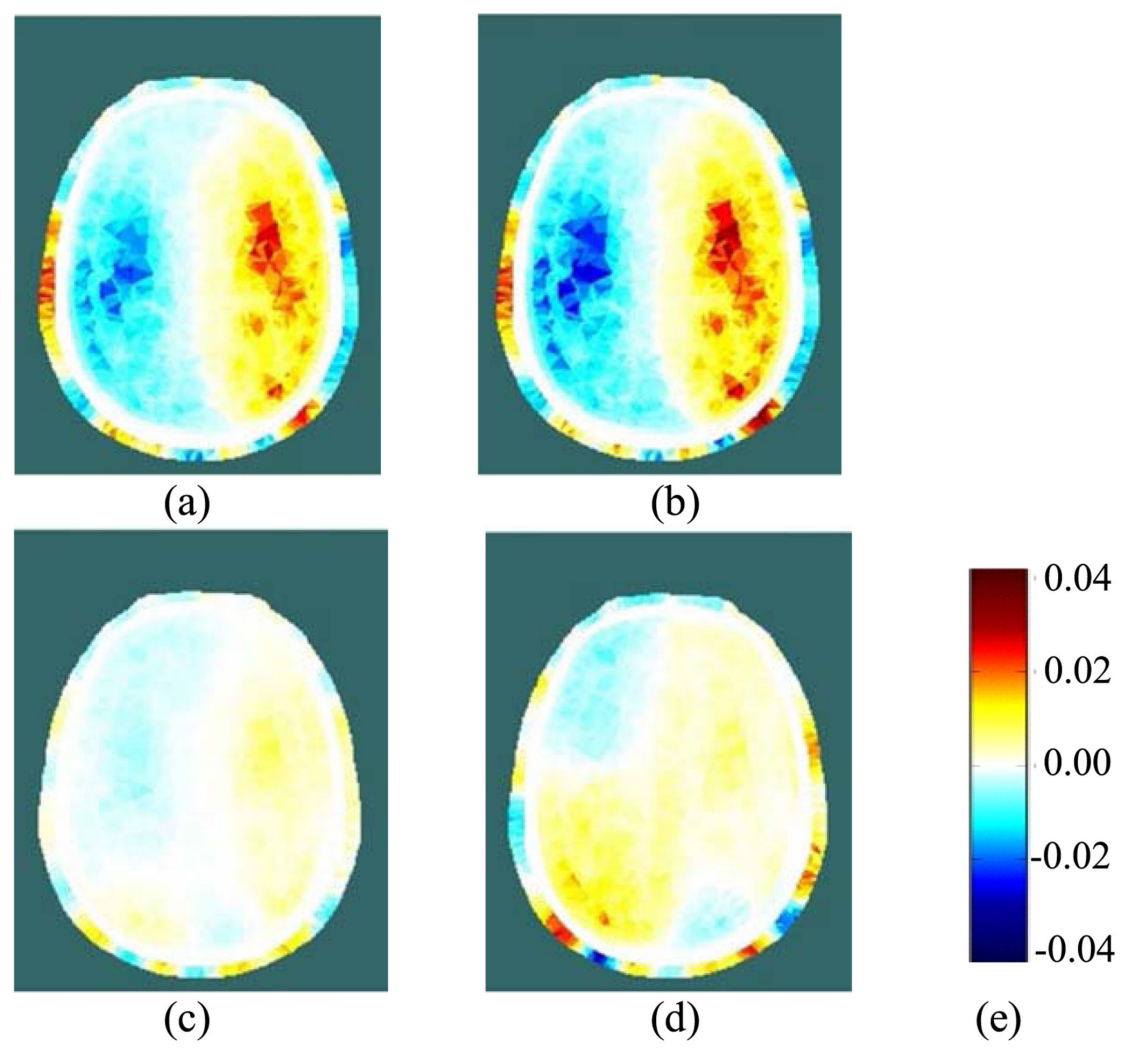
(a) and (b): Reconstructed EIT images during LEFT carotid artery occlusion (the first two blue-shaded periods in Fig. 9); (c) and (d): Reconstructed EIT images after relief of the occlusion (the last two blue-shaded periods in Fig. 9); (e): colour legend, depicting the change in conductivity, in S/m.

**Fig. 11:**
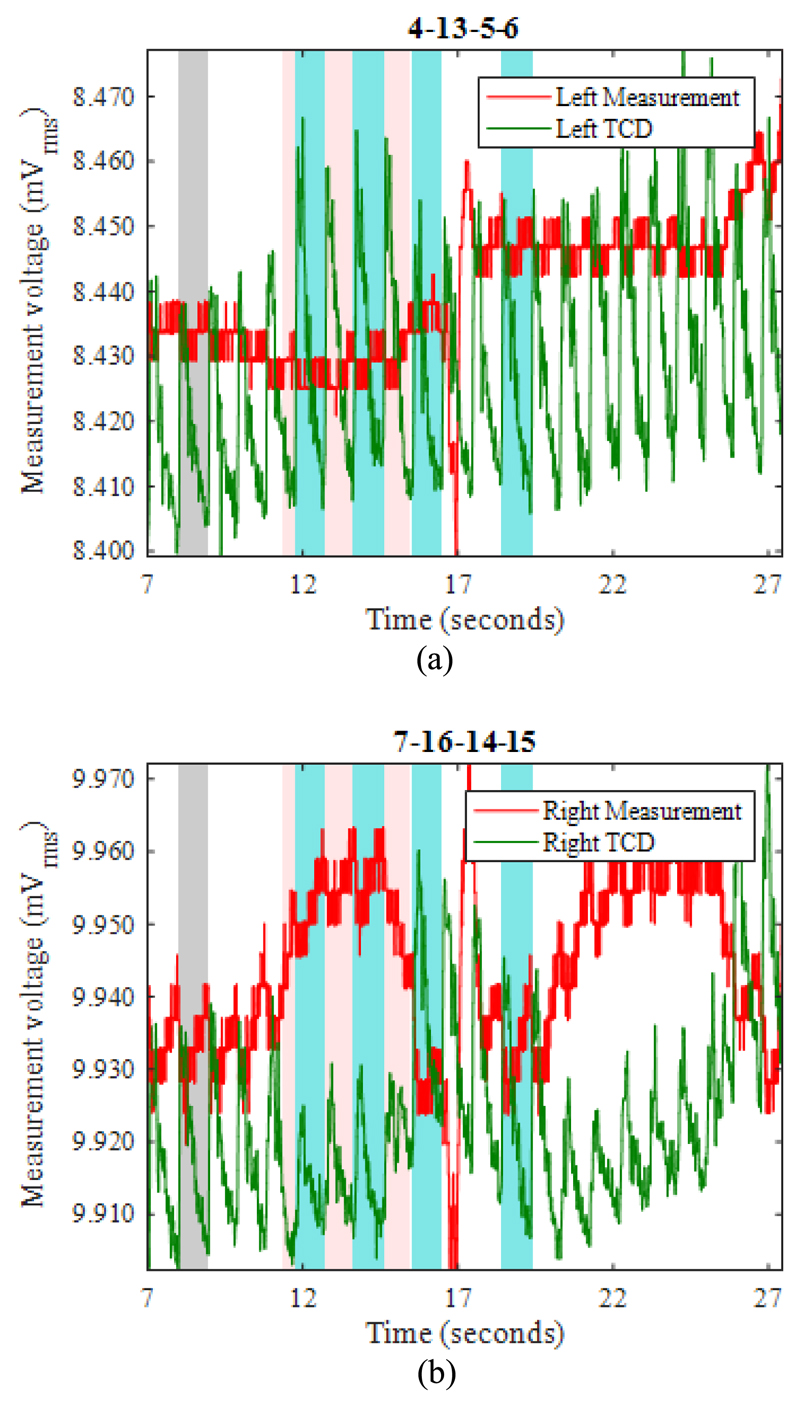
For RIGHT carotid artery occlusion during the period 10.9-15.0s (shaded pink), EIT measurements (red) and TCD measurements (green), recorded near (a) the left ear, and (b) the right ear. Grey shading shows the reference period for EIT image reconstruction.

**Fig. 12:**
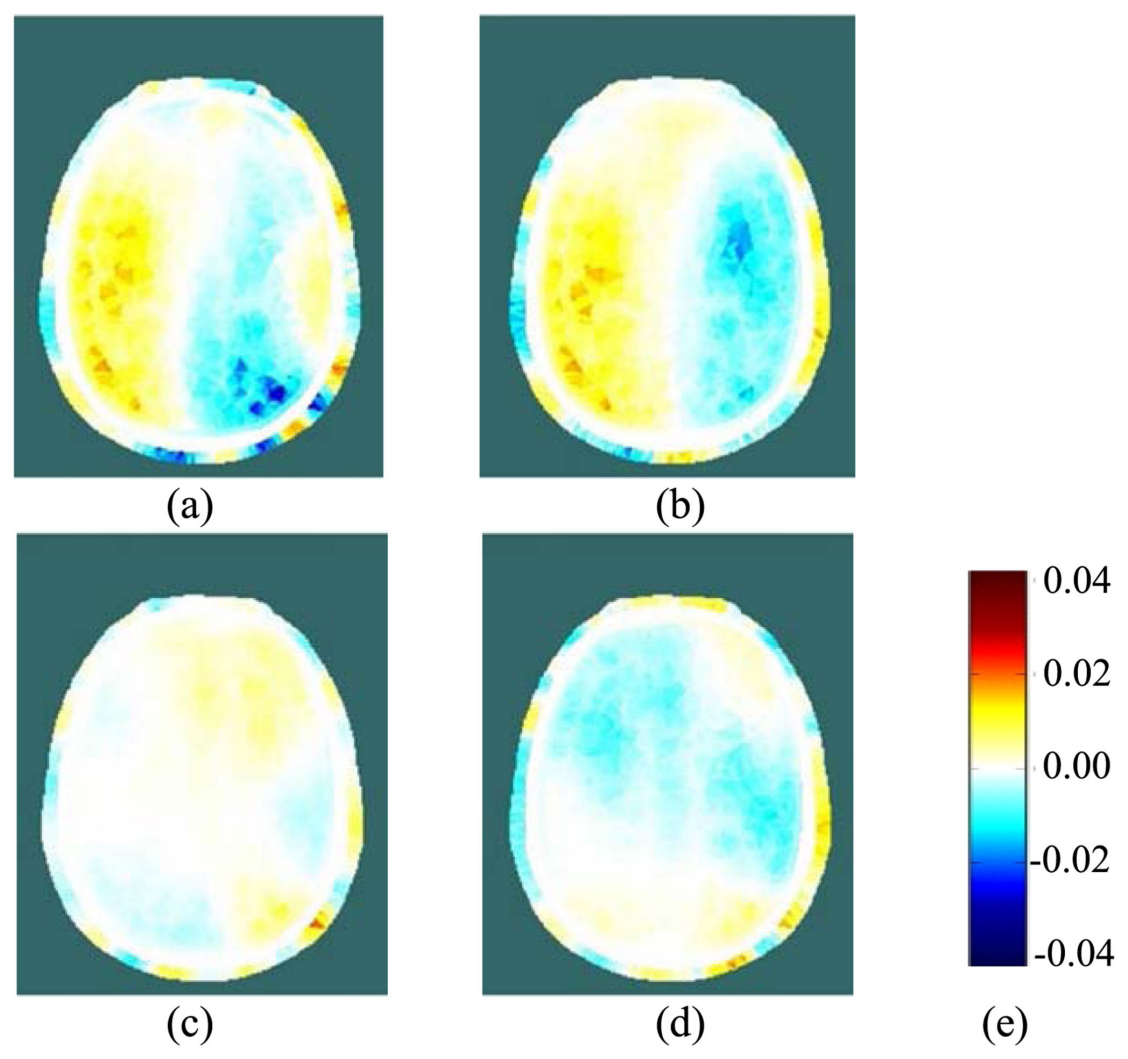
(a) and (b): Reconstructed EIT images during RIGHT carotid artery occlusion (the first two blue-shaded periods in Fig. 11); (c) and (d): Reconstructed EIT images after relief of the occlusion (the last two blue-shaded periods in Fig. 11); (e): colour legend, depicting the change in conductivity, in S/m.

Using EIT data averaged over complete REG cycles (shaded in blue in Fig. 9(a)-(b)), difference images were reconstructed for the change in conductivity relative to the average for the cardiac cycle immediately preceding the start of the occlusion period (shaded in grey). Within the set of reconstructed images (Figs. 10 and 12) for each THR trial, the colour legend is uniform. During the occlusion period, the reconstructed images in Figs. 10(a)-(b) and Figs. 12(a)-(b) show a marked reduction in conductivity on the occluded side, whilst the conductivity on the non-occluded side increases. The stark left-right contrast disappears after the occlusion is relieved, as shown in Figs. 10(c)-(d) and Figs. 12(c)-(d). Similar trends are observed clearly in the reconstructed images for 13 out of the 17 THR trials; the four trials where the reconstructed images do not correspond with the majority trend are conspicuous by the fact that they were all on the same volunteer subject and taken during the same measurement session. Considering the entire 3-dimensional dataset of images for all 17 tests, the peak local magnitude of the conductivity change of the grey/white matter regions during occlusion (relative to the pre-occlusion period) is typically 20%, far exceeding the steady-state REG variation observed in III.B.

## IV Discussion

Various reviews have been published in order to compare different EIT systems for biomedical applications, such as [37]. Shi *et al.* [15] have compared the performance of several EIT systems applied to the human head in particular. With its combination of high frame rate and excellent noise rejection, as discussed in sub-sections II.A and II.B, the fEITER system is unique in the published literature, even though it uses only the single current excitation frequency of 10 kHz.

Image reconstruction is dependent on the use of a reliable head model. For three of the five tissue types in the model used here, conductivity values are taken from the literature. To set the two most sensitive tissue conductivity values, *viz.* those of the scalp and skull, it was essential to carry out a global fit analysis [25] of fEITER data to the 208k–element head model (Table II). An important practical advantage of the image reconstruction method of II.D is the use of a non-subject-specific model (II.C) that provides robust agreement with forward predictions from long-term average measurements.

Practical image reconstruction using such a complex head model, combined with over 500 measurements for each frame of data, is enormously challenging. Fundamentally, it is enabled by the new non-linear method described in sub-section II.D above, which can cope with substantial model error.

Computational implementation of the method is achieved by using the Regularised Newton-Krylov GMRes technique also described in II.D, with 200 Krylov subspaces. The phantom results presented in sub-section III.A show that the novel reconstruction process is able to locate a 20 mm dia. blood inclusion at an SNR attainable by both variants of the fEITER instrument. The resilience of the reconstruction method to modelling error is key to the successful imaging shown in Fig. 3 and the middle rows of Fig. 4. This success increases confidence in the results obtained in human trials (III.B and III.C).

In more than 50 volunteers, the REG waveform was revealed by the most sensitive tetrapolar measurement configurations of fEITER Mk. I [8]. With fEITER Mk. II, the REG-related signal variations, ranging from a few *μ*V up to several tens of *μ*V, are much better resolved, and found to be present in all measurements (section III.B). The absence of any other discussion of the REG phenomenon in the EIT literature is remarkable. Fabrizi *et al.* [38], using the so-called KHU Mark 1 system operating at 12 fps, noted *“harmonics of about 1 Hz when applied on a human subject”,* which they attributed to imperfections in the instrumentation system.

There has been much discussion in the REG literature, e.g. [9], [10], regarding its dependence on intra-cranial versus extra-cranial processes, with a consensus that, compared with bipolar measurements, tetrapolar REG signals have greater dependence on intra-cranial processes and can track CBF [11]. The EIT images reconstructed from the 509 tetrapolar REG measurements per frame recorded with fEITER Mk. II, and illustrated in Figs. 7 and 8 above, exhibit substantial regions of conductivity change in the grey and white matter. The rate of change in the images from Fig. 7(a) to Fig. 7(c) illustrates the benefit of temporal resolution in the region of tens of fps. These findings merit further study with a statistically larger sample of volunteers. Clearly, in any EIT measurements of the head over periods of a few seconds or less, it is essential to account for signal variations attributable to REG.

For image reconstruction of fEITER Mk. I data from the short (~5s) THR trials reported in section III.C above, we have taken great care to average our EIT measurements over whole REG cycles. The resulting 3-dimensional images of conductivity difference (relative to the resting state) display a systematic and marked left-right contrast in response to left or right carotid artery occlusion, as illustrated in Figs. 10 and 12. Such images are observed clearly in 13 out of 17 trials conducted, with the remaining four attributable to a set-up error in one volunteer. The observed trends in conductivity variation, and their marked left-right lateral behavior, correspond closely with the expected blood flow behavior [12], [13] and with TCD data collected simultaneously.

During long-term (60s) common carotid artery occlusion on human subjects, Shi *et al.* [15] report 2-D EIT measurements at 50 kHz current excitation, achieving 1 fps image rate. They present 10 reconstructed images during each occlusion, with some evidence of a systematic difference in response to left occlusion versus right. It is difficult to compare their prolonged hemodynamic intervention with the effects of the much shorter occlusions used in our work.

## V Conclusion

The fEITER system reported here achieves a unique combination of high imaging speed (100 fps) and measurement sensitivity (SNR up to 95 dB in human trials, with excellent CMRR). Using a novel nonlinear 3D reconstruction method, EIT imaging of human cerebral hemodynamics using scalp-mounted electrodes has been demonstrated. The reconstruction method does not use a subject-specific model. Simulations show that it is resilient against modelling error and provides good localization of included conductivity contrasts (equivalent to blood) for measurement SNR of 70 dB or better.

For > 50 resting human volunteers, trans-impedance measurements using fEITER Mk. I display the REG waveform, synchronized with the cardiac cycle. For a resting human volunteer measured by Mk. II, EIT imaging of the REG process reveals ~1% tidal variation in cerebral conductivity. This variation shows considerable left-right symmetry and cycle-to-cycle repeatability. We believe that the resulting reconstructed images of conductivity difference in the white and grey matter during the REG cycle represent the first time-resolved images of cerebral perfusion due to the normal cardiac cycle. The intervals over which significant changes occur in these images are short, suggesting a minimum EIT frame rate of 25 fps for such studies.

EIT measurements during THR are highly correlated with TCD data. Using data from fEITER Mk. I, EIT images during the THR process (for measurements averaged over the REG cycle) show large changes in conductivity (~20%), far exceeding the steady-state variation. The images show strong left-right asymmetry, reflecting the application of the carotid artery occlusion. Similar behaviour was observed in 13 out of 17 trials, with the remaining 4 traceable to a single experimental error. We believe that these are the first 3D images to be observed during the THR procedure.

Taken together, these results provide compelling evidence that both steady-state and evoked variations in cerebral hemodynamics are observable using state-of-the-art EIT instruments and algorithms. The challenge now is to demonstrate the collection of clinically useful data.

## Figures and Tables

**Table I:** Current Patterns (CPs)

CP	Sites	CP	Sites
1	1-30	11	4-13
2	1-27	12	8-17
3	22-26	13	7-13
4	9-18	14	20-24
5	7-16	15	6-14
6	3-12	16	1-31
7	1-28	17	1-10
8	8-12	18	2-11
9	5-15	19	21-25
10	1-29	20	19-23

**Table II:** Head Tissues: Assigned Conductivity Values, And Number Of Elements

Tissue type	Conductivity (S/m)	Number of elements
Scalp	0.584	67,861
Skull	0.0084	45,502
CSF	1.802	26,712
Grey matter	0.2849	45,375
White matter	0.2556	22,861

**Table III:** Localization Error With New Image Reconstruction Method (mm)

SNR	MIDDLE	FRONT	LEFT	RIGHT
∞	17.3	16.3	22.8	26.8
70 dB	16.4 ± 4.0	16.2 ± 1.4	22.3 ± 3.3	27.9 ± 2.5
50 dB	32.4 ± 14.6	40.8 ± 21.7	34.5 ± 14.6	36.9 ± 13.3

(Mean ± Standared Deviation, except in the no-noise case)
